# Higher-order assemblies in immune signaling: supramolecular complexes and phase separation

**DOI:** 10.1007/s13238-021-00839-6

**Published:** 2021-04-09

**Authors:** Shiyu Xia, Zhenhang Chen, Chen Shen, Tian-Min Fu

**Affiliations:** 1 Department of Biological Chemistry and Molecular Pharmacology, Harvard Medical School, and Program in Cellular and Molecular Medicine, Boston Children’s Hospital, Boston, MA 02115 USA; 2 Department of Biological Chemistry and Pharmacology, The Ohio State University, Columbus, OH 43210 USA; 3 The Ohio State University Comprehensive Cancer Center, Columbus, OH 43210 USA

**Keywords:** higher-order assembly, phase separation, signalosome, cGAS, inflammasome, TCR, BCR, TLR, RLR, TNFR, death domain, immune signaling

## Abstract

Signaling pathways in innate and adaptive immunity play vital roles in pathogen recognition and the functions of immune cells. Higher-order assemblies have recently emerged as a central principle that governs immune signaling and, by extension, cellular communication in general. There are mainly two types of higher-order assemblies: 1) ordered, solid-like large supramolecular complexes formed by stable and rigid protein-protein interactions, and 2) liquid-like phase-separated condensates formed by weaker and more dynamic intermolecular interactions. This review covers key examples of both types of higher-order assemblies in major immune pathways. By placing emphasis on the molecular structures of the examples provided, we discuss how their structural organization enables elegant mechanisms of signaling regulation.

## Introduction

The immune system is typically categorized into innate immunity and adaptive immunity. Innate immunity is the first line of immune defense and recognizes general patterns of pathogen or danger signals to evoke immune defense, while adaptive immunity refers to antigen-specific immune response that involves the activation of T cells and B cells. Both innate immunity and adaptive immunity have evolved intricate signaling networks to defend against pathogens or sterile dangers.

A fundamental question in the field is how immune signaling networks make sensitive response to pathogens or danger signals while avoiding over-activation to induce autoimmune diseases. Recent studies have revealed that higher-order assembly, by which biomolecules cluster into large structures of defined or irregular shapes, provides not only a common theme in innate and adaptive immunity, but also elegant mechanisms of sensitivity control and signal transduction (Yin et al., 2015; Du and Chen, 2018; Courtney et al., 2018).

Formation of higher-order assemblies can be achieved in mainly two ways. First, through stable protein-protein interactions, proteins may form supramolecular complexes with solid-like behaviors and defined shapes. These supramolecular complexes are generally termed signalosomes. Second, phase-separated, more dynamic, and liquid-like droplets can form by weaker multivalent interactions. The process through which such condensation occurs is called liquid-liquid phase separation (LLPS).

**Figure 1:**
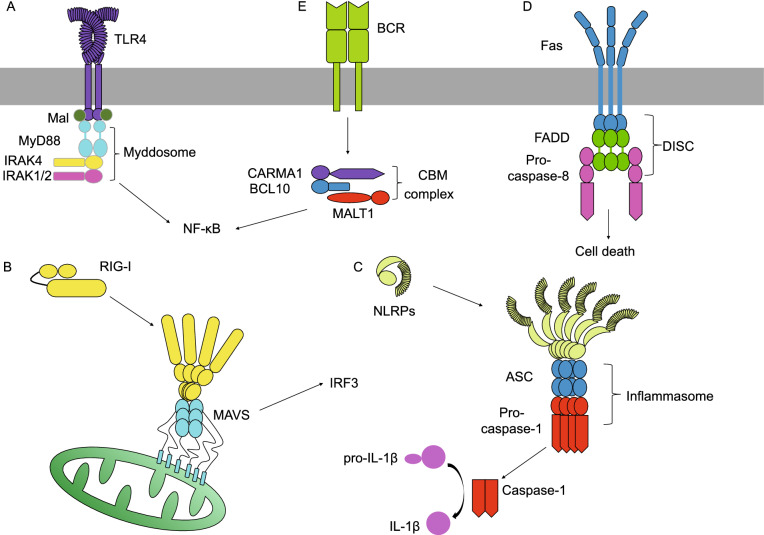
**Immune signaling pathways that involve death domain-containing proteins**. (A) The myddosome forms downstream of TLR4 via DD-DD interaction. (B) In an antiviral signaling pathway, RIG-I and MAVS assemble into a complex through CARD-CARD interaction. (C) PYD-PYD and CARD-CARD interactions enable the assembly and activation of an ASC-dependent inflammasome. (D) The Fas receptor, a member of the TNFR superfamily, initiates DISC assembly through DD-DD and DED-DED interactions. (E) A B-cell receptor signaling pathway via the CBM signalosome formed by CARD-CARD interaction

Examples of the first type of higher-order assemblies include those formed by members of the death domain (DD) superfamily. Found in many cellular receptors, adaptors, and effectors, DDs are protein-protein-interaction domains that mediate numerous host defense pathways in response to pathogen or danger signals. These pathways include Toll-like receptors (TLRs) signaling, RIG-I-like receptors (RLRs) signaling, inflammasomes signaling, tumor necrosis factor receptors (TNFRs) signaling, as well as B cell receptor (BCR) signaling (Fig. 1). The DD superfamily comprises four subfamilies known as DD, death effector domain (DED), caspase recruitment domain (CARD), and Pyrin domain (PYD) (Park et al., 2007a). Despite divergence in their primary sequence, the families share a conserved six-helix bundle structure, with minor variations in the lengths and orientations of the helices. Through oligomerization, DDs can form higher-order assemblies known as signalosomes for signal transduction. For instance, the C-terminal cytoplasmic portion of death receptor Fas contains a DD, which oligomerizes upon Fas ligand (FasL) engagement to interact with the DD of Fas-associated protein with death domain (FADD), thereby passing signals downstream (Wang et al., 2010).

Equally intriguing is the second type of higher-order assemblies mediated by LLPS, which represents a new concept of macromolecular organization in cells (Brangwynne et al., 2009; Banani et al., 2017). A hallmark of cells, in particular eukaryotic cells, is that biochemical reactions are spatially and temporally controlled. One way to achieve such control is that eukaryotic cells have many membrane-enclosed organelles to separate reactions from one another. Another way is the formation of membraneless biomolecular condensates mediated by LLPS. Rapid progress in the field has provided ample examples of physiological processes mediated by LLPS, including growth factors-mediated cell signaling, cytoskeleton assembly, transcription, chromatin organization, pathogenesis of neurodegenerative diseases, and immune response (Molliex et al., 2015; Murakami et al., 2015; Patel et al., 2015; Su et al., 2016; Du and Chen, 2018; Sabari et al., 2018; Case et al., 2019; Gibson et al., 2019; Guo et al., 2019; Huang et al., 2019). A key principle governing LLPS is multivalency, which is enabled by many mechanisms including the presence of multiple modular domains or motifs and intrinsic-disorder regions (IDRs) (Li et al., 2012; Han et al., 2012). Functionally, in addition to regulating the spatial organization of biochemical reactions, LLPS accelerates the reactions by increasing the local concentration of reactants. LLPS also contributes to noise reduction and robust response to true signals. Examples of LLPS that we discuss here are cyclic GMP-AMP synthase (cGAS)-stimulator of interferon genes (STING) signaling in innate immunity and T-cell receptor (TCR) signaling in adaptive immunity (Su et al., 2016; Du and Chen, 2018).

## Structures and intermolecular interactions of death domains

**Figure 2:**
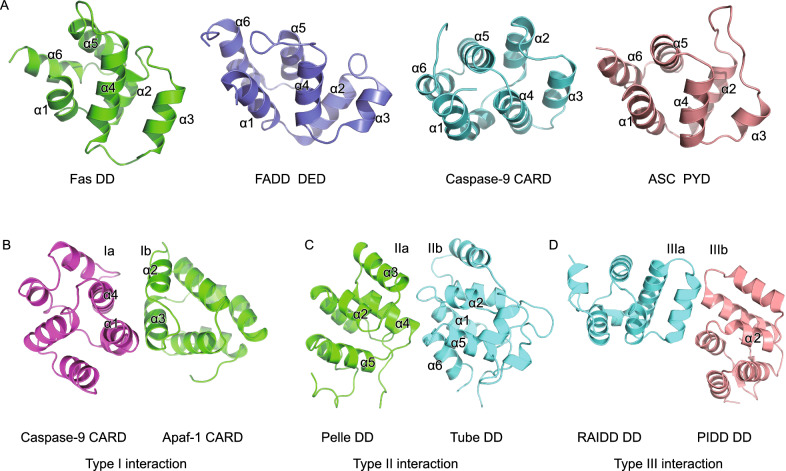
**Death domain structures and their interactions**. (A) Structures of Fas DD (PDB ID: 1DDF), FADD DED (PDB ID: 2GF5), caspase-9 CARD (PDB ID: 3YGS), and ASC PYD (PDB ID: 3J63) show a characteristic six-helix bundle organization conserved in the DD superfamily. (B) Type I interaction revealed by the crystal structure of the Apaf-1 CARD-caspase-9 CARD complex (PDB ID: 3YGS). (C) Type II interaction uncovered by the crystal structure of the Pelle DD-Tube DD complex (PDB ID: 1D2Z). (D) Type III interaction defined by the crystal structure of the RAID DD-PIDD DD complex (PDB ID: 2OF5)

The DD superfamily, including DD, DED, CARD, and PYD, feature a compact six-helix bundle structure arranged in an antiparallel fashion, although the lengths and orientations of the helices can be slightly different (Fig. 2A). First revealed by solution NMR, the structure of the Fas DD shows an amphipathic α-helical bundle with hydrophobic residues from each helix lining the core (Huang et al., 1996). Helices α1 and α2 reside at the center whereas α3 through α6 are peripherally located. Later structural studies have unveiled similar six-alpha-helical arrangements in FADD DED (Carrington et al., 2006), caspase-9 CARD (Qin et al., 1999), and apoptosis-associated speck like protein containing a caspase recruitment domain (ASC) PYD (Lu et al., 2014).

During immune response, DDs often assemble into oligomers or polymers through three types of interactions, establishing a molecular basis for protein-protein interaction and signal transduction. The X-ray crystal structure of apoptotic protease activating factor 1 (Apaf-1) CARD in complex with caspase-9 CARD defined Type I interaction, namely the asymmetric interaction between the basic concave surface formed by helices α1 and α4 (Type Ia surface) and the acidic convex surface formed by helices α2 and α3 (Type Ib surface) (Qin et al., 1999) (Fig. 2B). Later, the crystal structure of Pelle DD in complex with Tube DD defined Type II interaction, mediated mainly by α2-α3, α4-α5 loops (Type IIa surface) and α1-α2, α5-α6 loops (Type IIb surface) (Xiao et al., 1999) (Fig. 2C). Besides the 1:1 asymmetric Type I and II interactions revealed by the two dimeric complex structures, the PIDDosome structure uncovered Type III interaction between α3 (Type IIIa surface) and α1-α2 and α3-α4 loops (Type IIIb surface) (Park et al., 2007b) (Fig. 2D). In the PIDDosome structure, all three types of asymmetric interactions together mediate DD oligomerization, which turns out to be a common theme for the DD superfamily and plays crucial roles in cell death and immune signaling. Recently, the cryo-EM revolution enabled high-resolution structural determination of several helical filaments formed by members of DD superfamily via Type I, II, and III interactions (Lu et al., 2014). Interestingly, concrete biochemical and structural evidence to date exists only for interactions among DDs of the same subfamilies—for example, between two CARDs or between two PYDs, but not between a CARD and a PYD—although it has been reported that cross interactions may occur (Vajjhala et al., 2015).

As a primer, structural studies have revealed common themes that apply to DD assembly. First, DDs assemble into oligomers with helical symmetry; second, DD assembly is a nucleated hierarchical polymerization process; third, DD assembly is mediated by three different types of protein-protein interactions; fourth, DD helical oligomers increase the local concentration of kinases or caspases to facilitate their activation. The higher-order assemblies of the DD superfamily represent a new paradigm for signal transduction and a structural basis for threshold responses in immune signaling.

## TLR and RLR signaling

**Figure 3:**
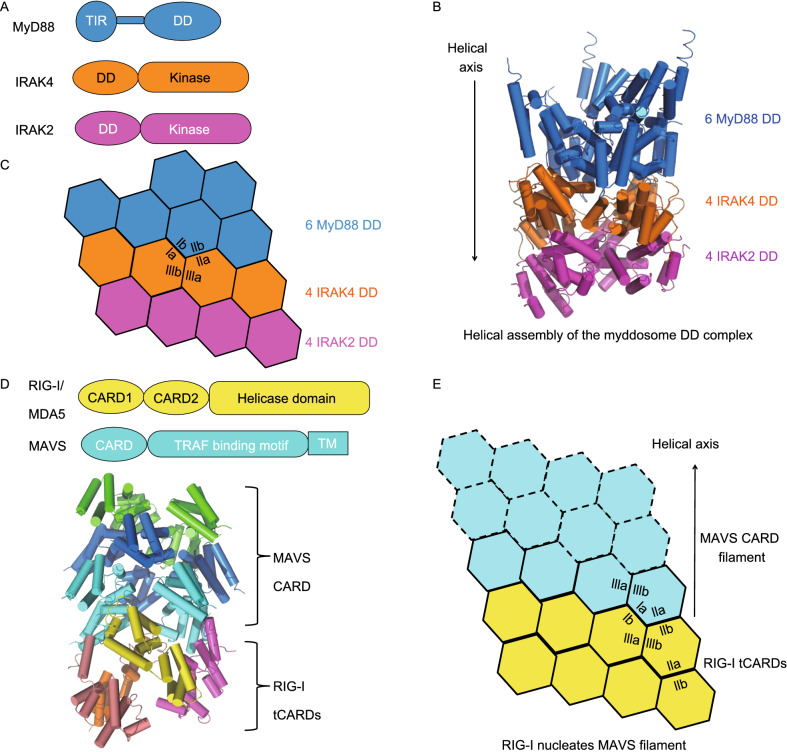
**TLR and RIG-I signaling pathways governed by death domain assembly**. (A) Domain architecture of myddosome components MyD88, IRAK4, and IRAK2. (B) Crystal structure of the myddosome DD complex (PDB ID: 3MOP). (C) Helical assembly of the myddosome DD complex with interfaces denoted. (D) Domain architecture of RIG-I, MDA5, and MAVS and the cryo-EM structure of the MAVS CARD-RIG-I tCARDs complex (PDB ID: 4P4H). (E) Helical assembly of the MAVS CARD filament nucleated by RIG-I tCARDs

As a class of pattern recognition receptors (PRRs), TLRs detect a variety of pathogen-associated molecular patterns (PAMPs) outside of the cells or inside cellular compartments, including flagellin, profilin, lipopolysacharrides (LPS), single-stranded RNA (ssRNA), and double-stranded RNA (dsRNA) (Kawai and Akira, 2006). TLRs have N-terminal successive leucine-rich repeats (LRRs) that serve to bind the PAMPs. At their C-termini, there is a Toll/IL-1 receptor (TIR) domain responsible for recruiting cytosolic adapter proteins such as myeloid differentiation factor 88 (MyD88) and MyD88-adapter-like (MAL), which also have a TIR domain (Fig. 3A). In addition to the TIR, MyD88 contains a DD that in turn recruits more downstream proteins such as IL receptor-associated kinase 1/2 (IRAK1/2) and 4 (IRAK4), which contain DDs and kinase domains, via DD-DD interaction (Ferrao et al., 2012). The MyD88-IRAK4-IRAK2 complex assembled by DD interaction is termed a myddosome. Finally, the IRAKs recruit and phosphorylate downstream effector molecules in order to activate NF-κB.

The crystal structure of a myddosome DD complex, consisting of 6 DDs from MyD88, 4 DDs from IRAK4, and 4 DDs from IRAK2, reveals a novel hierarchical and helical assembly mechanism (Lin et al., 2010) (Fig. 3B and 3C). The 14 DDs assemble into a left-handed helical oligomer, with shape and charge complementarity formed at each turn. However, it was unknown how the DDs of MyD88 subunits come into proximity to initiate myddosome assembly. Recently, cryo-EM structures revealed that the TIR domains of TLRs, MAL, and MyD88 polymerize into filaments, which serve as molecular platforms that bring several MyD88 subunits together (Ve et al., 2017). Collectively, these structures provide a mechanistic picture of TLR signaling, where TIR domain-mediated filament formation and DD-mediated myddosome assembly co-occur to drive the pathway forward.

RLRs represent another class of PRRs that are located in the cytoplasm and particularly active in the innate immune defense of epithelial and myeloid cells (Loo and Gale, 2011). In contrast to TLRs, which recognize extracellular or endosomal ligands, RLRs such as retinoic acid-inducible gene I (RIG-I) and melanoma differentiation-associated protein 5 (MDA5) recognize viral dsRNA that has entered the cytosol. RIG-I is a sensor of dsRNA with 5’ triphosphate (5’-ppp), whereas MDA5 detects long viral dsRNA (Kato et al., 2006; Myong et al., 2009). The structural motif critical for dsRNA binding is a DExD/H box helicase domain at the C-termini of RLRs (Kowalinski et al., 2011; Jiang et al., 2011; Luo et al., 2011; Wu et al., 2013a). In addition to the helicase domain, RIG-I and MDA5 both contain N-terminal tandem CARDs (tCARDs) (Fig. 3D). A model exists that dsRNA binding to the helicase domain causes a conformational change in RIG-I or MDA5 so that the tCARDs are exposed to recruit a downstream adapter by CARD-CARD interaction. The downstream adapter for RLRs is mitochondrial antiviral-signaling protein (MAVS), an N-terminal CARD-containing protein crucial for mediating NF-κB and IRF3 activation (Kawai et al., 2005; Meylan et al., 2005; Seth et al., 2005; Xu et al., 2005). Other than RIG-I and MDA5, a third RLR is LGP2, which shares the helicase domain but does not contain the CARD. Despite a previously assumed inhibitory role due to the lack of the signaling domain CARD, LGP2 was shown to increase the initial rate of MDA5-dsRNA interaction and enhance MDA5-mediated antiviral signaling (Bruns et al., 2014). The molecular mechanism of the cooperation between MDA5 and LGP2 remains elusive.

Similar to the MyD88-IRAK4-IRAK2 myddosome DD complex structure, the crystal structure of RIG-I tCARDs in complex with MAVS CARD and the cryo-EM structure of the MAVS CARD filament support helical assembly as a common mode of signal propagation in both TLR and RLR signaling (Wu et al., 2014) (Fig. 3D and 3E). Zooming in at the end of the filament, RIG-I tCARDs form a tetrameric platform that recruits MAVS CARD by Type I, II, and III interactions, a process commonly referred to as nucleation. Nucleated polymerization into filamentous structures appears as a unified mechanism of DD assembly, which we will revisit in inflammasomes, TNFR signaling, and the CBM signalosome.

## Inflammasome signaling and regulation

Within many types of immune cells, intricate supramolecular complexes termed inflammasomes are activated by diverse microbial and damage-associated signals to trigger immune response. Inflammasome activation ultimately leads to proteolytic activation of the pore-forming protein gasdermin D (GSDMD), release of proinflammatory cytokines such as interkleukin-1β (IL-1β) and IL-18, and pyroptosis (Xia, 2020; Xia et al., 2020).

There are canonical and noncanonical inflammasomes. Canonical inflammasomes, such as the nucleotide-binding domain, leucine-rich-containing family, PYD-containing protein 3 (NLRP3) inflammasome, the nucleotide-binding domain, leucine-rich-containing family, CARD-containing protein 4 (NLRC4) inflammasome, and the absent in melanoma 2 (AIM2) inflammasome, contain sensors of various signals including flagellin, extracellular ATP, K^+^ efflux, and cytosolic dsDNA (Hornung et al., 2009; Lightfield et al., 2011; Zhao et al., 2011; Munoz-Planillo et al., 2013). While canonical inflammasomes recruit and activate caspase-1, the noncanonical inflammasome relies on direct activation of murine caspase-11 (and human orthologs caspase-4 and -5) by cytosolic LPS (Shi et al., 2014).

**Figure 4:**
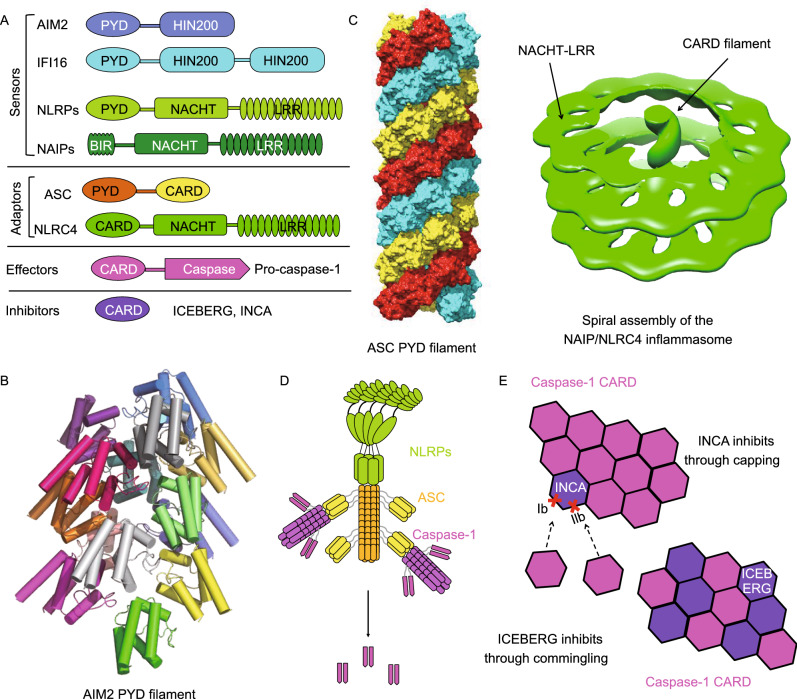
**Death domain-mediated inflammasome signaling and regulation**. (A) Domain organization of inflammasome components. (B) Cryo-EM structure of the AIM2 PYD filament (PDB ID: 6MB2). (C) Cryo-EM structure of the ASC PYD filament (PDB ID: 3J63) and cryo-electron tomography (cryo-ET) structure (PDB ID: 5AJ2) of the NAIP/NLRC4 inflammasome reveal helical assembly of PYD and CARD. (D) A model of NLRP inflammasome assembly mediated by PYD-PYD interaction between NLRP and ASC and CARD-CARD interaction between ASC and caspase-1. (E) Mechanisms of INCA and ICEBERG as inhibitors of CARD helical assembly during inflammasome activation

Recently, the assembly mechanisms of the three canonical inflammasomes mentioned above have been elucidated by structural and biochemical investigations (Jin et al., 2012; Lu et al., 2014; Diebolder et al., 2015; Hu et al., 2015; Lu et al., 2015; Zhang et al., 2015) (Fig. 4A–D). While the AIM2 and NLRP3 inflammasomes rely on the adapter protein ASC to activate caspase-1, the NLRC4 inflammasome directly engages and activates caspase-1. The ASC-dependent inflammasomes share a nucleated polymerization mechanism, where AIM2 and NLRP3 serve as nucleators for the polymerization of ASC through PYD-PYD interaction (Fig. 4B and 4C). It should be noted that although AIM2 PYD can assemble into a filament, the oligomerization of NLRP3 PYD is mediated by its NACHT domain (Lu et al., 2014). As an adaptor protein, ASC contains an N-terminal PYD and a C-terminal CARD, which are responsible for interaction with upstream PYD-containing sensors and downstream CARD-containing effectors (Fig. 4A). Both PYD and CARD of ASC are able to assemble into filaments with helical symmetry (Lu et al., 2014; Li et al., 2018). Different from the ASC-dependent inflammasomes, the helical assembly of the NLRC4 inflammasome is initiated through the activation of the sensor and nucleator NAIP by bacterial ligands (Diebolder et al., 2015; Zhang et al., 2015). In this spiral structure, NLRC4 CARD forms a filament at the center of the spiral, whereas the NACHT and LRR domains assemble into disk-like structures along the CARD filament axis (Fig. 4C). Sharing similar architecture, both ASC and NLRC4 CARD filaments recruit downstream caspase-1 through CARD-CARD interaction (Fig. 4D). Polymerization of caspase-1 CARD into filaments locally concentrates caspase-1 and induces the dimerization and auto-processing of caspase-1 into its active p10 and p20 fragments, which then cleave pro-IL-1β, pro-IL-18, and GSDMD to elicit inflammation and pyroptosis.

Given that cooperativity in nucleated polymerization facilitates rapid filament assembly, inflammasome activation exhibits an all-or-none behavior. It is therefore crucial that cells have evolved elaborate strategies to curb inflammasome activation when necessary. Recent structural studies highlighted two DD filament inhibitors – inhibitor of CARD (INCA) and ICEBERG (Lu et al., 2016). Known as CARD-only proteins (COPs), INCA and ICEBERG both contain one single CARD (Fig. 4A). However, they function by distinct mechanisms (Fig. 4E). ICEBERG interferes with the interaction between caspase-1 CARD and other CARD-containing proteins through commingling. In contrast, INCA prevents caspase-1 CARD polymerization into filaments through a capping mechanism. A closer inspection at subunit oligomerization interfaces reveals that INCA is defective in Type Ib and Type IIb, two of the six interfaces required for CARD-CARD interaction and filament formation. Other known inhibitors of DD assembly include PYD-only proteins ASC2 and POP2, albeit unclear molecular mechanisms (Natarajan et al., 2006; Ratsimandresy et al., 2017).

## A TNFR pathway

**Figure 5:**
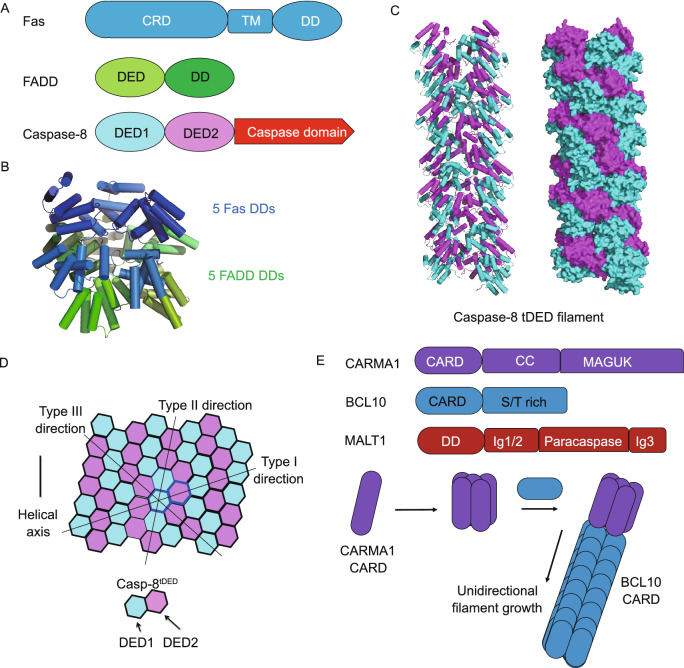
**Death domain signaling in Fas and CBM signalosome pathways**. (A) Domain architecture of DISC components including receptor Fas, adaptor FADD, and effector caspase-8. (B) Crystal structure of the Fas DD-FADD DD complex (PDB ID: 3OQ9) displays helical assembly similar to the MyD88-IRAK4-IRAK2 DD complex described above. (C) Cryo-EM structure of caspase-8 tDEDs (PDB ID: 5L08) shows helical assembly into filaments. (D) Extensive inter-subunit interactions in caspase-8 tDED filaments. (E) Domain architecture of CBM signalosome components and a nucleated polymerization model of CARMA1 CARD-BCL10 CARD filaments

The TNFR superfamily comprises type-I transmembrane receptors that respond to TNF-like ligands and plays crucial roles in numerous physiological processes including lymphoid development, immunity, cellular homeostasis, and apoptosis. Certain TNFR superfamily members, such as the Fas receptor and TNFR1, contain DDs in their intracellular portions, which can initiate cell death signaling (Fig. 5A). Upon FasL engagement, Fas DD recruits adapter protein FADD, which is composed of a DD and a DED, through DD-DD interaction. FADD further recruits caspase-8 through DED-DED interaction to form a ternary complex known as the death-inducing signaling complex (DISC), which facilitates the dimerization and activation of caspase-8.

Crystallographic and cryo-EM studies of the Fas DD-FADD DD complex revealed asymmetric oligomers of 5 to 7 Fas DDs and 5 FADD DDs (Wang et al., 2010). In the crystal structure, 5 Fas DDs and 5 FADD DDs form a two-layered structure that resembles the myddosome helical assembly (Fig. 5B). Based on quantitative western blots and mass spectrometry, caspase-8 is about 7- to 9-fold more abundant than FADD at the DISC, an observation that hints at a signal amplification function of DD assembly (Dickens et al., 2012; Schleich et al., 2012). Consistently, caspase-8 also robustly polymerizes into filaments with helical symmetry due to the presence of tandem DEDs (tDEDs) at its N-terminus (Shen et al., 2015; Fu et al., 2016) (Fig. 5C). Similar to other DD assemblies, caspase-8 tDED filaments utilize all three types of interactions (Fig. 5D). However, compared to other filaments formed by single domains, caspase-8 tDED filaments harbor more interactions, such that each type II or type III interface can each engage two different interfaces. The quasi-equivalent interactions in type II and type III interfaces are achieved through charge-charge complementarity, which is critical for the assembly and regulation of caspase-8 tDED filaments. Further structural modeling and biochemical assays showed that DISC assembly is a hierarchical, unidirectional process (Fu et al., 2016).

## The CBM signalosome in BCR signaling

Downstream of the B-cell receptor, CARM1, BCL10, and MALT1 assemble into a ternary complex known as the CBM signalosome to mediate NF-κB activation. CARMA1 is composed of an N-terminal CARD, a central coiled-coil domain, and a C-terminal MAGUK domain. Upon activation, CARMA1 recruits BCL10 through CARD-CARD interaction, after which the C-terminal Ser/Thr-rich domain of BCL10 interacts with the Ig1/2 domain of MALT1 to form the CBM signalosome (Fig. 5E). Together with the crystal structure of CARMA1 CARD, cryo-EM structure of the BCL10 CARD filament and time-lapse confocal microscopy provide insights into the assembly process of the CBM signalosome complex (Qiao et al., 2013; David et al., 2018). Nucleated by CARMA1 CARD, BCL10 CARD unidirectionally polymerizes into filaments through Type I, II, III interactions, a highly unified mechanism of assembly for the DD superfamily.

## cGAS-STING signaling

**Figure 6:**
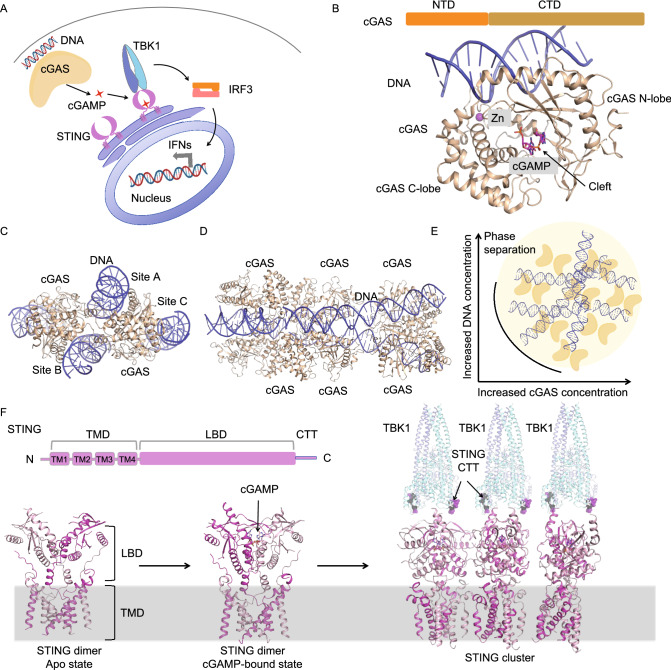
**Higher-order assemblies in cGAS-STING signaling**. (A) Overview of key molecules in cGAS-STING signaling. (B) Crystal structure of monomeric cGAS in complex with DNA and 2’3’-cGAMP (PDB ID: 4K9A). (C) Crystal structure of dimeric cGAS in complex with DNA (PDB ID: 4LEZ) showing three DNA binding sites in cGAS. Site A and site B contribute to the formation of the 2:2 cGAS-DNA complex. Site C serves as a third interaction site between cGAS and DNA, which is important for phase separation. (D) Crystal structure of oligomeric cGAS bound to long DNA ligands (PDB ID: 5N6I). (E) Liquid-liquid phase separation formed by cGAS and DNA ligands. (F) Mechanisms of cGAMP-induced STING oligomerization and subsequent recruitment and activation of TBK1. STING is composed of an N-terminal transmembrane domain, a ligand-binding domain (LBD), and a C-terminal tail (CTT). Apo STING exists as a constitutive dimer on the ER membrane (PDB ID: 6NT6). Upon cGAMP engagement, dimeric STING undergoes large conformational changes and forms clusters (PDB ID: 6NT7, 6NT8). Activated STING exposes its CTT for the recruitment of TBK1 (PDB ID: 6NT9). The STING oligomer brings many TBK1 molecules into proximity for their trans-autophosphorylation and activation

cGAS is a major cytosolic DNA sensor in innate immunity (Sun et al., 2013). Upon DNA engagement, cGAS produces a second messenger called cGAMP, which binds and activates the downstream effector protein STING (Ablasser et al., 2013; Gao et al., 2013a; Sun et al., 2013; Wu et al., 2013b) (Fig. 6A). Upon activation, STING recruits TANK-binding kinases 1 (TBK1), which then phosphorylates interferon regulatory factor 3 (IRF3), leading to the dimerization and nucleus translocation of IRF3 for the transcriptional induction of type I IFNs and inflammatory cytokines to trigger immune response (Ablasser and Chen, 2019; Zhang et al., 2020) (Fig. 6A). Accumulating evidence has shown that this cGAS-STING signaling pathway plays vital roles in many physiological and pathophysiological processes, including anti-virus immunity, autophagy, cellular senescence, and inflammatory diseases (Li et al., 2013b; Gray et al., 2015; Chen et al., 2016; An et al., 2017; Yang et al., 2017; Gui et al., 2019).

Structural investigation of cGAS and STING has revealed the molecular principles governing the assembly and function of cGAS and STING. cGAS is composed of a disordered N-terminal domain and a C-terminal catalytic domain (Fig. 6B), which belongs to a large family of nucleotidyltransferase (NTases) responsible for catalyzing the transfer of nucleoside monophosphates. The catalytic domain of cGAS adopts a bilobal fold, which contains an N-terminal lobe that is composed of two helices and a twisted beta sheet for catalysis and a C-terminal lobe composed of a helical bundle for mediating DNA binding and dimerization (Gao et al., 2013a; Civril et al., 2013) (Fig. 6B). The large cleft between the two lobes is responsible for binding substrates (Fig. 6B). Opposite to the substrate-binding cleft is a surface groove that can bind DNA (Fig. 6B). cGAS utilizes positively charged residues to engage the sugar-phosphate backbone of DNA, which explains the lack of sequence specificity in DNA recognition by cGAS (Civril et al., 2013; Gao et al., 2013a). DNA binding triggers the re-organization of the cGAS catalytic site to enhance substrate accessibility and thereby promotes the synthesis of cGAMP (Civril et al., 2013; Gao et al., 2013a).

Although DNA ligands shorter than 20 bp are sufficient to trigger cGAMP production, longer DNA is required for the full activation of cGAS *in vitro* and cGAS signaling *in vivo* (Luecke et al., 2017). Structures of DNA-bound cGAS showed two DNA-binding sites in cGAS that allowed the formation of a 2:2 DNA-cGAS complex (Li et al., 2013a; Zhang et al., 2013) (Fig. 6C). More strikingly, structures of long DNA ligands in complex with cGAS revealed higher-order assembly of cGAS dimers along the DNA ligands (Andreeva et al., 2017) (Fig. 6D). The assembly process appears cooperative given that the initial 2:2 cGAS-DNA complex allows rearrangement of the two DNA molecules in a parallel fashion (Andreeva et al., 2017).

More recent findings revealed that long DNA can further induce LLPS by cGAS (Du and Chen, 2018) (Fig. 6E). In cells, LLPS is fundamental for the formation of membraneless cellular compartments, including the stress granules, germ granules, P bodies, Cajal bodies, nuclear speckles, and the nucleolus (Hyman et al., 2014; Shin and Brangwynne, 2017). Studies have revealed that multivalency of macromolecules is the key factor that drives LLPS, which is also true in the case of cGAS. As mentioned above, cGAS contains a disordered N-terminal domain and a C-terminal catalytic domain, both of which contain DNA-binding sites. The high density of positively charged residues in the N-terminal domain and the three identified DNA-binding sites in the C-terminal domain provide the structural basis for multivalent interaction between cGAS and DNA. Indeed, biochemical assays showed that cGAS and DNA form liquid condensates in a concentration-dependent manner (Du and Chen, 2018). LLPS by cGAS and DNA was shown to enhance the enzymatic activity of cGAS for the synthesis of 2’3’-cGAMP (Du and Chen, 2018). By contrast, a more recent study revealed that cGAS-DNA LLPS does not directly control 2’3’-cGAMP synthesis (Zhou et al., 2021). Instead, cGAS-DNA LLPS serves to resist suppression by multiple negative immune regulators including barrier-to-autointegration factor 1 (BAF1) and the cytosolic exonuclease Trex1 (Zhou et al., 2021). As a major suppressor of DNA sensing, Trex1 degrades cytosolic dsDNA to prevent autoimmune disease (Kavanagh et al., 2008). In this context, cGAS-DNA LLPS protects DNA from degradation by limiting the entry of Trex1 into the cGAS-DNA LLPS as well as suppressing Trex1 exonuclease activity within the LLPS (Zhou et al., 2021). Together, current studies reveal at least three biological implications for the formation of cGAS-DNA LLPS. First, long DNA is more potent than short DNA in triggering cGAS signaling because long DNA has a higher valency than short DNA and a lower threshold to induce the formation of LLPS. Second, the formation of cGAS-DNA phase separation provides a new mechanism for balancing DNA-dependent immune activation and suppression. Third, phase separation allows the cGAS signaling pathway to mount digital immune defense against infection while avoiding autoimmune reactions to low concentrations of self-DNA. Further study is required to fully discern the functional roles of the cGAS-DNA LLPS *in vivo*.

As the downstream effector protein of cGAS, STING is located at the endoplasmic reticulum membrane and composed of an N-terminal domain containing four transmembrane helices, a cytosolic ligand-binding domain (LBD) for recognizing cGAMP, and a C-terminal tail (CTT) for recruiting downstream TBK1 (Fig. 6F). Earlier crystal structures showed that the LBD of STING assembles as a constitutive dimer for binding cGAMP (Ouyang et al., 2012; Yin et al., 2012; Gao et al., 2013b; Zhang et al., 2013). cGAMP engagement induces the rotation of the two LBDs toward each other to form a closed conformation, which is coupled to the activation of STING for downstream signaling. Recently, a cryo-EM study of full-length STING and its complex with cGAMP revealed two unexpected features that are critical for STING activation (Shang et al., 2019) (Fig. 6F). First, upon cGAMP engagement, the LBD of STING undergoes a 180° rotation relative to the TM domain. Second, a loop on the side of LBD is switched to a less extended conformation for mediating the STING dimer-dimer interaction, leading to the formation of STING oligomers. The C-terminal tails of dimeric STING recruit and activate TBK1. The oligomerization of STING is critical for the activation of TBK1 because the STING oligomers cluster multiple TBK1 dimers to facilitate trans-autophosphorylation of the activation loop, which is a critical step in TBK1 activation (Zhang et al., 2019; Zhao et al., 2019).

## TCR signaling

**Figure 7:**
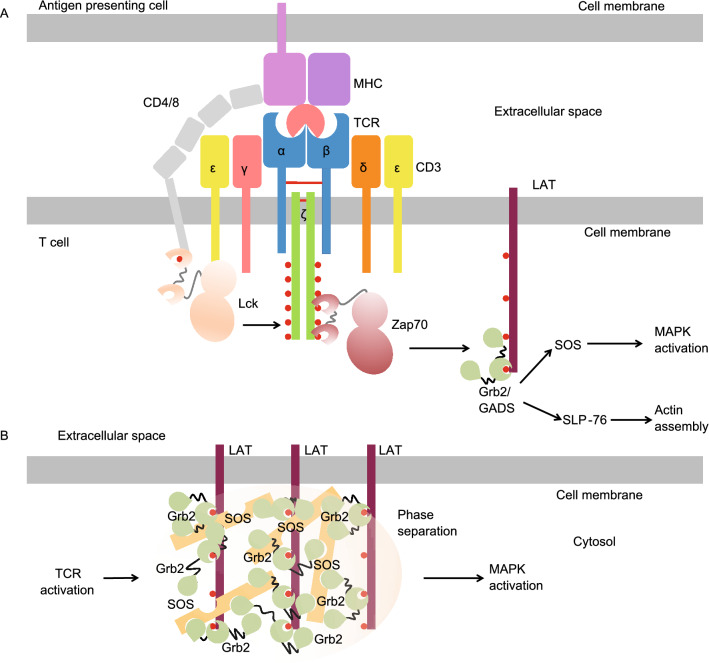
**Phase separation in TCR signaling**. (A) Overview of the TCR signaling pathway. (B) Schematic of liquid-liquid phase separation that is assembled by LAT, Grb2, and SOS

TCR is responsible for detecting antigens that are presented by major histocompatibility (MHC) molecules. In cells, TCR is composed of αβ or γδ heterodimers, CD3ɛδ, CD3ɛγ, and a ζ chain homodimer in a 1:1:1:1 stoichiometry (Courtney et al., 2018) (Fig. 7A). Here we use αβ-TCR as an example to illustrate the activation mechanism of TCR. In the TCR complex, each ζ chain has a cytosolic ITAM motif with multiple tyrosine residues, which can be phosphorylated by Lck kinase that is brought to proximity to TCR by CD4 or CD8, which are co-receptors of TCR (Shaw et al., 1990) (Fig. 7A). The phosphorylated motifs within the TCR complex recruit the Zap70 kinase, leading to its phosphorylation and subsequent activation, which initiates TCR signaling (van Oers et al., 1994; Thill et al., 2016) (Fig. 7A).

As T cells require fewer than 10 agonistic MHC molecules or even only 1 single agonistic MHC molecule to trigger a full response (Irvine et al., 2002), it is clear that signal amplification plays a critical role in T cell activation. A key mechanism for signal amplification is provided by the LAT (linker for activation of T-cells) signaling hub. Activated Zap70 phosphorylates the adaptor protein LAT for the recruitment of additional adaptor proteins such as Grb2 and GADS, which will further engage SOS or SLP-76 (Courtney et al., 2018) (Fig. 7A). These proteins in the LAT signaling hub will activate downstream effector proteins which mediate mitogen-activated protein kinase (MAPK) activation and actin polymerization (Fig. 7A). Recent studies by *in vitro* reconstitution using supported lipid bilayers revealed that the LAT and downstream signaling proteins assemble into condensates by LLPS (Huang et al., 2016; Su et al., 2016) (Fig. 7B). The process involves multivalent interactions that are mediated by LAT, Grb2, and SOS (Su et al., 2016) (Fig. 7B). The micrometer-sized clusters of LAT and the associated proteins serve as a biochemical reaction center that facilitates MAPK activation and actin polymerization.

## Concluding remarks

Higher-order assemblies, in the forms of solid-like supramolecular complexes and phase-separated liquid droplets, represent a new paradigm for cell signaling. Different from the classical model of cell signaling that is triggered by conformational changes of receptors, higher-order assembly generally involves the polymerization of receptors, adaptors, and effectors into large structures of defined rigid shapes or into dynamic liquid-like condensates. These two types of higher-order assemblies share many features, such as concentration dependence, multivalency, as well as nucleated polymerization (Wu and Fuxreiter, 2016). A key difference between these two types of higher-order assemblies is that the solid-like supramolecular complexes are often stable and feature a unidirectional assembly process, while the liquid condensates are more dynamic and reversible. In some cases, liquid-like condensates can also convert into the more stable and solid-like state (Molliex et al., 2015; Murakami et al., 2015; Patel et al., 2015). It is likely that biological systems have evolved these two different types of higher-order assemblies to fine-tune the dynamics and reversibility of physiological processes.

Signaling by higher-order assemblies offers at least two advantages. First, the cooperative assembly process allows cells to make digital, threshold-like response. In other words, cells utilize this elegant mechanism to filter out biological noise and only respond to real signals that are strong and persistent. As immune signaling is a double-edged sword that may result in defense or autoimmunity, the immune system has evolved such an elegant mechanism that utilizes higher-order assembly to prevent unnecessary immune activation. Second, higher-order assemblies create a biochemical reaction center that significantly increases the local concentration of effector proteins, thereby enabling the proximity-driven protein activation and spatial regulation of cell signaling. These advantages may also manifest in cells that do not possess immune functions. We look forward to future discoveries that extend higher-order assemblies to a general principle in cell biology.
